# Extremely Efficient Catalysis of Carbon-Carbon Bond Formation Using “Click” Dendrimer-Stabilized Palladium Nanoparticles

**DOI:** 10.3390/molecules15074947

**Published:** 2010-07-20

**Authors:** Didier Astruc, Cátia Ornelas, Abdou K. Diallo, Jaime Ruiz

**Affiliations:** ISM, UMR CNRS N°5255, Université Bordeaux 1, 3305 Talence Cedex, France

**Keywords:** palladium, catalysis, nanoparticle, dendrimer, “click reaction”

## Abstract

This article is an account of the work carried out in the authors’ laboratory illustrating the usefulness of dendrimer design for nanoparticle palladium catalysis. The “click” synthesis of dendrimers constructed generation by generation by 1→3 C connectivity, introduces 1,2,3-triazolyl ligands insides the dendrimers at each generation. Complexation of the ligands by Pd^II^ followed by reduction to Pd^0^ forms dendrimer-stabilized Pd nanoparticles (PdNPs) that are extremely reactive in the catalysis of olefin hydrogenation and C-C bond coupling reactions. The stabilization can be outer-dendritic for the small zeroth-generation dendrimer or intra-dendritic for the larger first- and second-generation dendrimers. The example of the Miyaura-Suzuki reaction that can be catalyzed by down to 1 ppm of PdNPs with a “homeopathic” mechanism (the less, the better) is illustrated here, including catalysis in aqueous solvents.

## 1. Introduction

Catalysis by dendrimers appeared 20 years ago when Shell patented van Leeuwen’s work on the catalysis of CO/alkene polymerization. This study involved the comparison between mononuclear and star-shaped hexaphosphine-palladium catalysts. The star-shaped catalyst gave 3% fouling whereas the mono-palladium catalyst gave 50% fouling, which was already a positive dendritic effect [1,2]. Later, dendrimer catalysis was widely developed [3,4,5,6,7,8] for most known catalytic processes, the catalyst being covalently linked to the dendrimer frame [9,10,11,12,13,14,15,16,17]. Palladium catalysts are the most frequently used catalysts in synthesis [18], and this trend remains true for dendrimer catalysis [19,20]. Dendrimer catalysts indeed present the advantages of recyclability to a certain extent [21,22,23,24] and dendritic effects that can occasionally be positive [25,26,27,28,29,30,31,32,33,34,35], but experiences in our group have indicated that the concept is somewhat limited by the leaching of Pd [36,37,38,39]. Therefore, we have investigated the possibility of PdNP catalysis upon coordinating Pd^II^ to intradendritic nitrogen ligands. Triazolyl ligands have thus been introduced during dendritic construction upon “click” chemistry, followed by reduction to Pd^0^ atoms that agglomerate in PdNPs protected by the dendrimer frame. The concept of dendrimer-encapsulated nanoparticle catalysis had been introduced and developed since of the 90s by Crooks with polyamidoamine (PAMAM) dendrimers, and small dendrimers have also been shown to stabilize nanoparticles that are too large to fit in the dendrimer interior [40,41,42,43,44,45,46,47,48,49,50,51,52,53,54]. The catalytic reactions were carried out in organic solvents, water, supercritical CO_2_ (sc CO_2_), or fluorous/organic biphasic solvents. Besides PAMAM and poly(propyleneimine) (PPI) dendrimers that are commercially available, there has been only a few reports concerning the catalysis by dendrimer-stabilized nanoparticles, in particular with phenylazomethine dendrimers reported by the Yamamoto group [55,56,57,58].

## 2. Palladium Nanoparticle (PdNP) Catalysts in Carbon-Carbon Coupling Reactions

PdNP catalysis was developed by Beller *et al*. and Reetz *et al*. in 1996 [59,60], and this prolific area, including mechanistic issues in the Heck reaction, have been reviewed [61,62,63,64,65,66]. Aryl iodides and activated aryl bromides are easily coupled. Even aryl chlorides can couple, but much less selectively and in lower yields at temperatures of the order of 150 °C or higher. The source of PdNPs can be ligand free Pd complexes such as palladacycles, pincers, solid supports such as mesoporous silica, metal oxides, zeolites, hydroxyapatite, activated carbons, and organic polymers. In many examples, including polymer-stabilized PdNPs [67], it has been shown that leaching of Pd^0^ from the PdNPs (even temporarily and in very small amounts) into the solution provides the actual catalysts that are ligandless soluble mono- or biatomic palladium species [61,62,63,64,65,66].

Dendrimers are perfect macromolecules having a behavior related to that of polymers that have been long used for nanoparticle stabilization. The great advantage of dendrimers over polymers, however, is their perfect molecular definition and specific topology allowing molecular engineering that involves introducing a precise number of atoms into the PdNP layer after layer subsequent to the dendritic construction generation by generation [17,68,69,70].

## 3. Highly Efficient “Click”-Dendrimer-Encapsulated and Stabilized Pd Nanoparticle Pre-Catalysts

PAMAM and PIP dendrimers had not been specially designed for catalysis. Therefore, appropriate dendrimer design was necessary with a view to improved nanoparticle catalysis performance. We had reported arene-cored star [71] and dendrimer synthesis [72,73,74,75] by CpFe^+^-induced arene activation [76] involving nona-allylation of mesitylene following photolysis with visible light [77]. Generation growth with 1→3 connectivity [78,79] was carried out in a divergent way using selective hydrosilylation with HSiMe_2_CH_2_Cl, followed by nucleophilic substitution by a phenolate triallyl dendron producing dendrimers terminated by 3^n+2^ allyl branches (n = 0–7). [73,74,75] Variation of divergent growth included cross metathesis with acrylate functionalized dendron at the focal point [80] or “click” chemistry between a propargyl modified dendron at the focal point and dendritic cores terminated by azido groups [81]. The 1,2,3-triazolyl dendrimers obtained by “click” chemistry were ideal ligands for Pd^II^ [82] and Au^III^ [83].

**Figure 1:**
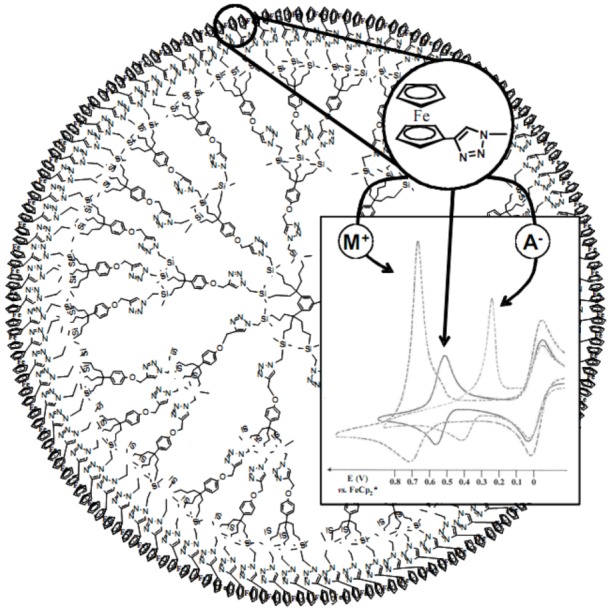
Cyclic voltammetry: recognition of both oxo-anions and transition-metal cationic acetonitrile complexes by a second-generation “click” ferrocenyl dendrimer.

When these click dendrimers were terminated by 1,2,3-triazolyl ligands connected to ferrocenyl (Fc) groups, the Fc groups were used as redox sensors for the recognition and titration of Pd^II^ ions. This electrochemical recognition was straightforward using cyclic voltammetry, because all the peripheral redox Fc groups appeared equivalent in a single wave that is both chemically and electrochemically reversible [84,85]. Various transition metal cations and oxo-anions (including ATP^2-^) could be sensed with selectivity, as monitored by the shift of the redox potential of the Fc wave. This titration technique was simple and useful to in order to determine the number of Pd^II^ cations encapsulated in the “click” dendrimers of various generations (Figure 1). [86]

**Figure 2:**
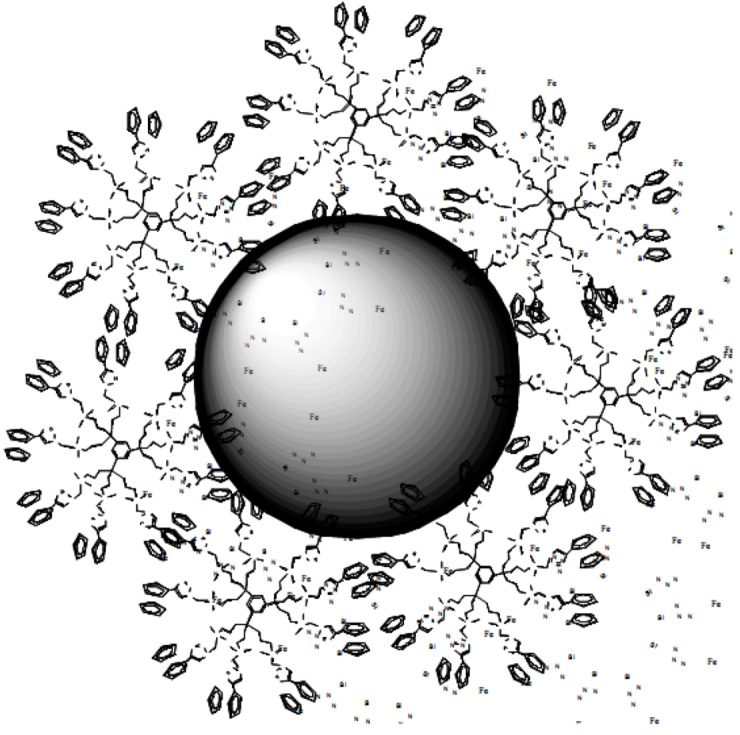
Pd nanoparticle surrounded and stabilized by several small G_0_ nonaferrocenyl dendrimers.

There are various known modes of coordination of Pd^II^ with triazole ligands, and the monohapto mode X-ray mode was confirmed by X-ray crystal structure determination [87]. Reduction of the G_1_ (27 Fc) and G_2_ (81 Fc) dendritic-Pd^II^ complexes using NaBH_4_ or methanol provided PdNPs for which the sizes, determined by TEM, corresponded to the theoretical number of Pd atoms according to the one-to-one stoichiometry determined by electrochemical titration of the Pd^II^ precursors. This result was indicative of intra-dendritic PdNP formation and encapsulation. On the other hand, the PdNPs formed from the G_0_ dendrimer (9 Fc) were large. This small dendrimer cannot encapsulate NPs, but stabilization is still occurring by locating dendrimer around the PdNPs (Figure 2). 

**Scheme 1:**
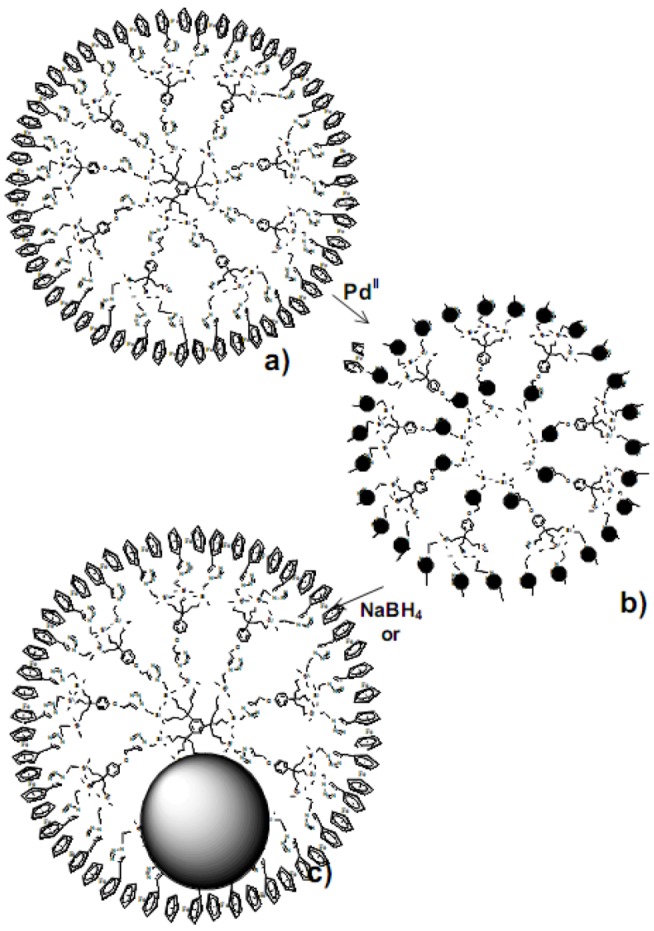
Synthesis of “click”-ferrocenyl dendrimer-encapsulated PdNPs.

The fact that these G_0_-PdNPS were large confirmed that the size was independent on the dendrimer size when the latter is too small. Thus the smallest PdNPs were those formed from the G_1_ dendrimer containing 27 Fc groups 36 triazolyl rings and encapsulating PdNPs that contained 36 Pd atoms (Scheme 1).

Selective hydrogenation of dienes to monoenes was readily achieved under ambient conditions for small dienes [88], but large steroidal dienes failed to react, in accord with their lack of ability to reach the PdNP surface. The rates (TOFs) and TONs of hydrogenation were all the larger as the PdNPs were smaller, as expected from previous results with polymer-stabilized PdNPs [67] according to a mechanism that involves mechanistic steps of the hydrogenation on the PdNP surface [88].

## 4. “Homeopathic” Catalysis of Miyaura-Suzuki C-C Coupling by “Click” Dendrimer-Stabilized PdNPs under Ambient Conditions

**Scheme 2:**
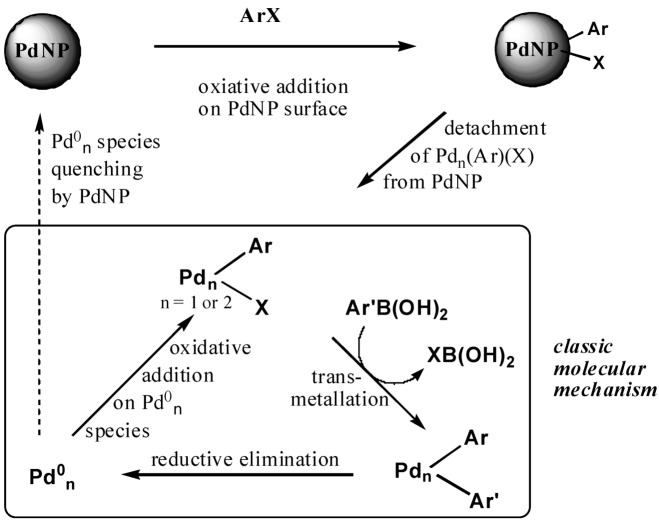
Leaching mechanism in the “homeopathic” catalysis of Suzuki C-C coupling at ambient temperature between PhI and PhB(OH)_2_ by “click” ferrocenyl dendrimer-stabilized PdNPs [64].

Whereas hydrogenation catalysis proceeds at the PdNP surface, as shown above, and therefore depends on the PdNP size, the catalysis of Miyaura-Suzuki C-C coupling [89] between PhI and PhB(OH)_2_ was carried out at room temperature and does not depend on the PdNP size and whether its stabilization is intra- or interdendritic. This shows that the dendrimer is not involved in the rate-limiting step of the reaction. The dendrimer-stabilized PdNPs work identically, whatever their size, and the TONs increase upon decreasing the amount of catalyst from 1% down to 1 ppm or upon dilution of the reaction solution. Thus, the efficiency of the catalyst is remarkable in homeopathic amounts (54% yield at 25 °C with 1 ppm equivalent of Pd atom, *i.e.*, TON = 540,000) and a quantitative yield is not even reached (75% yield) with 1% equivalent Pd atom. [90] The “homeopathic” catalysis was already observed for the Heck reaction at 150°C and was rationalized by de Vries on the basis of a leaching mechanism involving detachment of Pd atoms from the PdNP subsequent to oxidative addition of the organic halide PhI on the PdNP surface [64,66]. This mechanism is established for high temperature reactions due to decomposition of the Pd catalyst to naked PdNPs, but it is less expected for a room-temperature reaction. The ease of the room-temperature reaction must be due, however, to the lack of ligation onto the dendrimer-stabilized PdNPs that therefore can easily undergo oxidative addition of PhI at their surface, which provokes the leaching of Pd atoms. These isolated Pd atoms are apparently extraordinarily reactive in solution, because their do not bear ligands other than the very weakly coordinating solvent molecules. The limit in their efficiency lies in that they are trapped by their mother NP if the solution is moderately concentrated. This inhibiting trapping mechanism is all the less efficient as the catalyst is more diluted in the solution, therefore it is not efficient under extremely diluted solutions whereas it strongly inhibits catalysis at relatively high concentrations. It is likely that this concept can be extended to other PdNP-catalyzed C-C bond formation reactions (Scheme 2).

Analogous click-dendrimer-stabilized PdNP with other termini including sulfonate providing solubility in water were also active in aqueous media for hydogenation and Suzuki coupling reaction with high TOF and TON numbers [91], as were also related “click”-polymer-stabilized PdNPs [92].

**Scheme 3:**
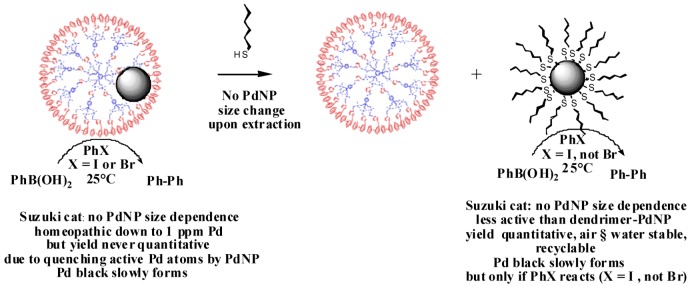
Extraction of “click”-dendrimer-encapsulated PdNPs from the dendrimer with hexathiol leading to hexanethiolate-PdNPs.

The G1-dendrimer-encapsulated PdNPs can be extracted by hexanethiol to yield a PdNP-cored hexanethiol star that also catalyze the Suzuki reaction under ambient conditions between phenylboronic acid and iodobenzene, but not bromobenzene, contrary to the G1-dendrimer-encapsulated PdNPs. PdNP-cored decanethiolate species were formerly found to be air and water stable and to be good catalysts for the latter Suzuki reaction. Thus, the thiolate ligands are not a poison for this catalysis, but the PdNP are not as free in the presence of the alkylthiolate ligands as in the dendrimer-stabilized PdNPs that are extremely active catalysts (Scheme 3) [93].

## 5. Conclusions and Prospects

Homogeneous PdNP catalysis is of interest, because the efficiencies and rates are, in many cases, considerably higher than those obtained with molecular catalysts for olefin hydrogenation and Miyaura-Suzuki reactions. The most difficult C-C coupling reactions involving non-activated aryl chlorides require very reactive molecular catalysts, however, although all catalysts decompose at high temperature to PdNPs that are also active in Heck coupling of aryl chlorides. The seminal work by Crooks’ group, [43,44,45] among others, had elegantly demonstrated multiple opportunities of PAMAM-dendrimer-encapsulated nanoparticle catalysis. PPI [94,95,96,97,98,99] of phenylazomethine [55,56,57,58] dendrimers have also offered related possibilities. Our contribution focused on the design of very active “click”-dendrimer-encapsulated and “click”-dendrimer-stabilized PdNP catalysts showing the key role of the intradendritic triazole ligands in these nanoreactors that worked in organic as well as aqueous solvents. This approach provided very active PdNP catalysts for C-C coupling reactions under ambient conditions and led to information concerning the leaching mechanism occurring in PdNP catalysis. Recent work has indicated that related leaching mechanisms may occur in other dendrimer-Pd-catalyzed reactions [100].
